# Selective Functional Disconnection of the Dorsal Subregion of the Temporal Pole in Schizophrenia

**DOI:** 10.1038/srep11258

**Published:** 2015-06-09

**Authors:** Lixue Xu, Wen Qin, Chuanjun Zhuo, Jiajia Zhu, Huaigui Liu, Xingyun Liu, Yongjie Xu, Chunshui Yu

**Affiliations:** 1 Department of Radiology and Tianjin Key Laboratory of Functional Imaging, Tianjin Medical University General Hospital, Tianjin 300052, China; 2 Tianjin Anding Hospital (Tianjin Mental Health Center), Tianjin City 300222, China; 3 Tianjin Anning Hospital, Tianjin City 300300, China

## Abstract

Although extensive resting-state functional connectivity (rsFC) changes have been reported in schizophrenia, rsFC changes in the temporal pole (TP) remain unknown. The TP contains several subregions with different connection patterns; however, it is not known whether TP subregions are differentially affected in schizophrenia. Sixty-six schizophrenia patients and 76 healthy comparison subjects underwent resting-state fMRI using a sensitivity-encoded spiral-in (SENSE-SPIRAL) imaging sequence to reduce susceptibility-induced signal loss and distortion. The TP was subdivided into the dorsal (TPd) and ventral (TPv) subregions. Mean fMRI time series were extracted for each TP subregion and entered into a seed-based rsFC analysis. Direct between-group comparisons revealed reduced rsFC between the right TPd and brain regions involved in language processing and multisensory integration in schizophrenia, including the left superior temporal gyrus, left mid-cingulate cortex, and right insular cortex. The rsFC changes of the right TPd in schizophrenia were independent of the grey matter reduction of this subregion. Moreover, these rsFC changes were unrelated to illness severity, duration of illness and antipsychotic medication dosage. No significant group differences were observed in the rsFC of the left TPd and bilateral TPv subregions. These findings suggest a selective (the right TPd) functional disconnection of TP subregions in schizophrenia.

Schizophrenia is a chronic, disabling mental disorder that has been considered a disconnection syndrome and that exhibits both anatomical and functional disconnections in multiple brain regions^1^. The most rostral portion of the temporal cortex, the temporal pole (TP), has been known to play an important role in language^2^,^3^, multisensory integration^4^,^5^, and social affective behaviours^6^; these behaviours have been found to be impaired in schizophrenia. Although two previous studies did not reveal significant grey matter volume (GMV) changes in the TP in schizophrenia^7^,^8^, other studies have reported significantly reduced GMV in the TP in patients with chronic schizophrenia^9^,^10^, in first-episode patients with schizophrenia^11^,^12^, and in young relatives at risk for schizophrenia^13^. Reduced GMV in the TP has been associated with deficits in formal thought disorder^14^ and theory of mind^15^ in schizophrenia. Structural abnormalities (thinner cortex) in the TP are even found in neonates who carry a risk gene for schizophrenia^16^. Schizophrenia patients also exhibit task-evoked activation changes in the TP during cognitive or emotional tasks^17^,^18^,^19^, and abnormal activation has even been observed in risk gene carriers^20^. Moreover, network analysis shows that schizophrenia patients exhibit significantly longer node-specific path lengths of the TP, indicating network connectivity impairment of the TP^21^. More importantly, several recent studies have provided evidence of abnormal resting-state functional connectivity (rsFC) of the TP in schizophrenia patients with visual hallucinations^22^, in first-episode patients with schizophrenia^23^, and in schizophrenia patients with or without neuroleptic treatment^24^. However, none of these studies used the TP as a seed region to fully elucidate the rsFC changes of the TP in schizophrenia.

Its functional diversity suggests that the TP may contain several subregions, which has been confirmed by two recent MRI-based parcellation studies that investigated the TP. Based on anatomical connection patterns, the TP is subdivided into the dorsal (TPd), medial (TPm) and lateral (TPl) subregions^25^. Based on rsFC patterns, the left TP is divided into the dorsal, ventromedial, medial, and anterolateral subregions^26^. These findings suggest that each TP subregion has a specific connection pattern and that these subregions may be involved in different functional networks. Investigation of subregional changes in the TP may provide the characteristic pattern of the rsFC impairment of the TP in schizophrenia, which may improve our understanding of the role of each TP subregion in the physiopathology of schizophrenia. Nevertheless, all previous research on schizophrenia has considered the TP as a single region, leaving TP subregional changes in schizophrenia largely unknown.

The lack of targeted functional MRI (fMRI) studies of the TP in schizophrenia can be largely attributed to the inherent limitations of the echo-planar imaging (EPI) technique. Most fMRI studies examine blood oxygenation level-dependent (BOLD) signals using the EPI technique, which inevitably causes susceptibility-induced signal loss and distortion in the TP region. This flaw also reduces our confidence in reliability of TP functional changes in schizophrenia derived from previous fMRI studies based on the EPI technique. To reduce susceptibility artefacts in air/tissue interfaces, a sensitivity-encoded spiral imaging (SENSE-SPIRAL) technique has been proposed to acquire fMRI data^27^. This technique can improve the fMRI quality of the TP and makes rsFC analysis of the TP more reliable than the conventional EPI sequence.

In this study, we aimed to use the SENSE-SPIRAL imaging technique to test the hypothesis that the rsFC of TP subregions is not uniformly impaired in schizophrenia.

## Methods and Materials

### Participants

A total of 98 in-patients with schizophrenia and 91 healthy comparison subjects were recruited for this study. Diagnoses for patients were confirmed using the Structured Clinical Interview for DSM-IV. The inclusion criteria included age (18–55 years), Chinese Han population, and right-handedness. The exclusion criteria included MRI contraindications, poor image quality, presence of a systemic medical illness or CNS disorder, history of head trauma, substance abuse within the last 3 months or a lifetime history of substance abuse or dependence. Additional exclusion criteria for the healthy comparison subjects included a history of any Axis I or II disorders, diagnosis of a psychotic disorder or a first-degree relative with a psychotic disorder. Four schizophrenia patients were excluded because of their oversized head motion (translational or rotational motion parameters more than 2 mm or 2°). In addition, 28 patients and 15 healthy comparison subjects were excluded because of distortion and signal loss in the TP region. A total of 66 schizophrenia patients and 76 healthy comparison subjects were finally included in further analysis (Table 1). The clinical symptoms of psychosis were quantified with the Positive and Negative Syndrome Scale (PANSS)^28^. This study was approved by the Medical Research Ethics Committee at Tianjin Medical University General Hospital, and after complete description of the study to the participants, written informed consent was obtained. The method was carried out in accordance with the approved guidelines.

### Image data acquisition

MRI was performed using a 3.0-Tesla MR system (Discovery MR750, General Electric, Milwaukee, WI, USA). Tight but comfortable foam padding was used to minimize head motion, and earplugs were used to reduce scanner noise. Sagittal 3D T1-weighted images were acquired by a brain volume sequence with the following parameters: repetition time (TR) = 8.2 ms; echo time (TE) = 3.2 ms; inversion time = 450 ms; flip angle (FA) = 12°; field of view (FOV) = 256 mm × 256 mm; matrix = 256 × 256; slice thickness = 1 mm, no gap; and 188 sagittal slices. Two sets of resting-state fMRI data were acquired. A gradient-echo single-shot EPI sequence was performed using the following parameters: TR/TE = 2000/45 ms; FOV = 220 mm × 220 mm; matrix = 64 × 64; FA = 90°; slice thickness = 4 mm; gap = 0.5 mm; 32 interleaved transverse slices; 180 volumes. A gradient-echo SENSE-SPIRAL (spiral-in) sequence was performed using the following parameters: TR/TE = 1400/30 ms; FA = 60°; 250 volumes; acceleration factor = 2. The FOV, matrix, slice thickness, gap, and slice number were the same as the EPI sequence. All slices were parallel to the AC-PC line. No particular adjustments for shimming or gradient moment correction were applied. During fMRI scans, all subjects were instructed to keep their eyes closed, to relax and move as little as possible, to think of nothing in particular, and to not fall asleep.

### GMV calculation

The GMV of each voxel was calculated using Statistical Parametric Mapping software (SPM8; http://www.fil.ion.ucl.ac.uk/spm/software/spm8/). The structural MR images were segmented into grey matter (GM), white matter and cerebrospinal fluid using the standard unified segmentation model. After an initial affine registration of the GM concentration map into Montreal Neurological Institute (MNI) space, GM concentration images were nonlinearly warped using diffeomorphic anatomical registration through the exponentiated lie algebra (DARTEL) technique^29^ and were resampled to 1.5-mm cubic voxels. The GMV of each voxel was obtained by multiplying the GM concentration map by the non-linear determinants derived from the spatial normalization step. Finally, GMV images were smoothed with a Gaussian kernel with a 6 × 6 × 6 mm^3^ full-width at half maximum (FWHM). After spatial preprocessing, the normalized, modulated, and smoothed GMV maps were used for statistical analysis.

### fMRI data preprocessing

Two sets of resting-state fMRI data were preprocessed using SPM8 using the same procedures. The first 10 volumes for each subject were discarded to allow the signal to reach equilibrium and the participants to adapt to the scanning noise. The remaining volumes were then corrected for the acquisition time delay between slices. All subjects’ fMRI data were within defined motion thresholds (translational or rotational motion parameters less than 2 mm or 2°). We also calculated framewise displacement (FD), which indexes volume-to-volume changes in head position^30^. There were no significant group differences in FD (t = 0.654, p = 0.514) between the schizophrenia patients (0.114 ± 0.063) and the healthy comparison subjects (0.108 ± 0.053). Because recent studies have reported that the signal spike caused by head motion significantly contaminates final resting-state fMRI results even after regressing out the realignment parameters^30^, we removed spike volumes if the FD of that specific volume exceeded 0.5. Several nuisance covariates (six motion parameters and the average BOLD signals of the ventricles, white matter and whole brain) were regressed out from the data. The datasets were band-pass filtered, with a frequency range of 0.01 to 0.08 Hz. Individual structural images were linearly coregistered to the mean functional image; then, the transformed structural images were segmented into GM, white matter, and cerebrospinal fluid. The GM maps were linearly coregistered to the tissue probability maps in MNI space. Finally the motion-corrected functional volumes were spatially normalized to MNI space using the parameters estimated during linear coregistration. The functional images were resampled into a 3 × 3 × 3 mm^3^ voxel. After normalization, all datasets were smoothed with a Gaussian kernel with a 6 × 6 × 6 mm^3^ FWHM.

### Definition of TP subregions

**Figure 1:**
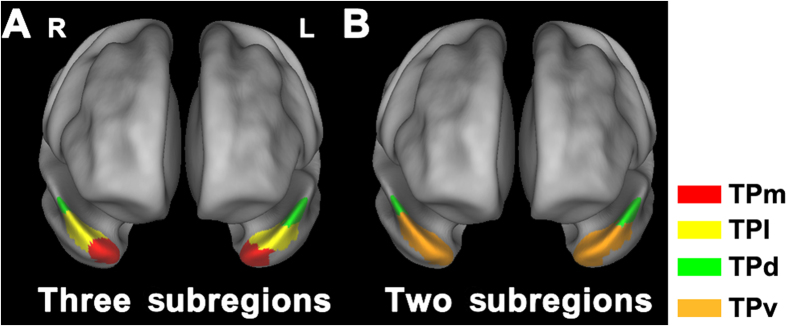
The maximal probability maps of subregions of the temporal pole (TP). (**A**) shows the maximal probability maps of the dorsal (TPd), medial (TPm) and lateral (TPl) subregions of the TP. (**B**) demonstrates the maximal probability maps of the dorsal (TPd) and ventral (TPv) subregions of the TP.

A previous study parcellated the TP into the TPd, TPm and TPl subregions based on anatomical connection patterns^25^. Initially, we defined a total of 6 subregions in the bilateral TP according to the maximal probability maps obtained in this study (Fig. 1A). However, the rsFC patterns of the TPm and TPl were very similar in both healthy comparison subjects and schizophrenia patients (Figures S1, S2 in Supplement 1), suggesting that they belonged to the same functional subregion of the TP. Therefore, we merged the TPm and TPl into a ventral subregion (TPv) (Fig. 1B). Finally, a total of 4 TP subregions were defined.

### Image quality assessments

TP image quality was compared between the EPI and SENSE-SPIRAL sequences with regard to signal intensity and distortion of the TP. The relative signal intensity (rSI) of each TP region (TPd, TPm and TPl bilaterally) was calculated by dividing the signal intensity of each region by the mean signal intensity of the whole-brain GM and was compared between the two imaging methods. The distortion severity was assessed by observing deviations of the normalized functional images from the structural images. As expected, the SENSE-SPIRAL sequence had better image quality; therefore, the fMRI data from this sequence were used to perform rsFC analysis. To further exclude the possible effect of signal loss on our results, we excluded subjects whose rSI in any TP subregion was lower than 0.5. Moreover, we also inspected the temporal signal-to-noise ratio (tSNR) of the TP subregions. The tSNR is defined as the mean of a voxel timecourse divided by its temporal standard deviation^31^. For each TP subregion in each individual, we calculated the tSNR of the motion-corrected and normalized fMRI timecourse^25^,^26^,^32^. Then, we compared the tSNR between healthy comparison subjects and schizophrenia patients.

### Independent component analysis (ICA)

To determine whether the TP subregions were involved in different “canonical” resting-state networks (RSNs), we performed an ICA analysis in healthy comparison subjects based on the realigned, normalized and smoothed SENSE-SPIRAL images using the group ICA (GICA) method. The detailed procedure has been described in our previous study^33^. Ten meaningful components were identified as “canonical” RSNs. Then, we performed a one-sample *t*-test to generate group masks for these RSNs (family-wise error (FWE)-corrected p < 0.05, two-tailed, cluster size > 30 voxels) (Figure S3 in Supplement 1). To investigate the involvement of each TP subregion in these “canonical” RSNs, we inspected the overlap between each RSN mask and each TP subregion.

### rsFC analysis

For individual datasets, Pearson’s correlation coefficients between the mean time series of each TP subregion and the time series of each voxel in other parts of the brain GM were computed and converted to *z* values using Fisher’s *r*-to-*z* transformation to improve normality. Each individual’s *z* values were then entered into a random-effect one-sample *t*-test in a voxel-wise manner to identify brain regions that showed significant positive correlations with each TP subregion. Then, a two-sample *t*-test was performed within the positive rsFC mask to quantitatively compare group differences in the rsFC of each TP subregion after controlling for age and gender. Multiple comparisons for these analyses were corrected using a false discovery rate (FDR) method (p < 0.05, two-tailed). We only focused on the positive rsFC networks because the biological meaning of negative rsFC networks is a matter of debate^34^,^35^. To exclude the possible effect of GMV on rsFC changes, we repeated the rsFC comparisons with the GMV of each TP subregion as an additional covariate. The same statistical threshold (p value) that was used in the original rsFC analysis was used in the repeated analysis.

### Node degree analysis

In graph theory, the degree of a node is defined as the number of edges linked to this node. Here, each TP subregion was treated as a node, and the significant functional connectivity between the node and each grey matter voxel in the brain (excluding the node itself) was treated as an edge. We calculated the degree of each node under different connectivity thresholds, ranging from 0 to 0.4 (0, 0.1, 0.2, 0.3, 0.4) Pearson’s correlation coefficients (r). A two-sample *t*-test was performed to compare group differences in node degree in each TP subregion. The significance level was set at p < 0.05 after Bonferroni correction for multiple comparisons.

### Validation analysis

Considering that the TP subregions extracted from the maximal probability maps may result in information overlap across subregions due to the normalization and smoothing preprocessing steps, we also defined the TP subregions using an alternative method (spheres with a radius of 6 mm centred at the centre of gravity of each TP subregion) to exclude the effect. We repeated our rsFC analyses to test whether the different methods used for seed definition would influence our results.

### Correlations between imaging and clinical parameters

To test whether the GMV and rsFC of the TP subregions with significant intergroup differences were correlated with the clinical variables, we extracted these imaging measures and calculated Spearman’s correlation coefficients between these imaging measures and the clinical parameters (i.e., PANSS score, duration of illness, and antipsychotic dosage). A value of p < 0.05 was considered significant.

## Results

### Image quality assessments of the TP subregions

TP signal intensity in four representative subjects is displayed in Figure S4 in Supplement 1. Images acquired with the SENSE-SPIRAL sequence exhibited less signal dropout than images acquired with the EPI sequence. The mean rSI of each TP subregion is shown in Table S1 and Figure S5A, 5B in Supplement 1. For all TP subregions, the mean rSIs derived from the SENSE-SPIRAL fMRI were greater than 0.5; however, the mean rSI of the left TPm derived from the EPI fMRI was less than 0.5. Using the structural images as a gold standard, the normalized functional images derived from the SENSE-SPIRAL fMRI exhibited less distortion in the TP and orbitofrontal cortex than those derived from the EPI fMRI (Figure S6 in Supplement 1). Therefore, the functional images derived from the SENSE-SPIRAL fMRI were used for the rsFC analysis. Moreover, we excluded subjects whose rSI in any region was lower than 0.5 to ensure the signal intensity of the TP was high enough. This procedure excluded 28 patients and 15 healthy comparison subjects. The mean rSI of each TP subregion of the remaining subjects is shown in Table S2 and Figure S5C, 5D in Supplement 1. We did not find any significant intergroup differences in rSI in any of the TP subregions (Table S3 in Supplement 1). We also did not find any significant group differences in the tSNR in any of the TP subregions except for the left TPv (t = 2.390, p = 0.018) (Table S4 in Supplement 1).

### Demographic and clinical characteristics of subjects

We finally included 66 schizophrenia patients (38 males; age: 33.0 ± 7.6 years) and 76 healthy comparison subjects (38 males; age: 33.0 ± 10.3 years). The demographic and clinical characteristics of these subjects are summarized in Table 1. There were no significant group differences in sex (χ^*2*^ = 0.815, p = 0.367) or age (t = −0.026, p = 0.980). To further exclude the effects of sex and age on our results, we considered these two variables as covariates of no interest throughout the rsFC analyses. Sixty-one patients were receiving atypical antipsychotics when performing the MRI examinations; five patients had never received any medications.

### The rsFC map of each group

**Figure 2:**
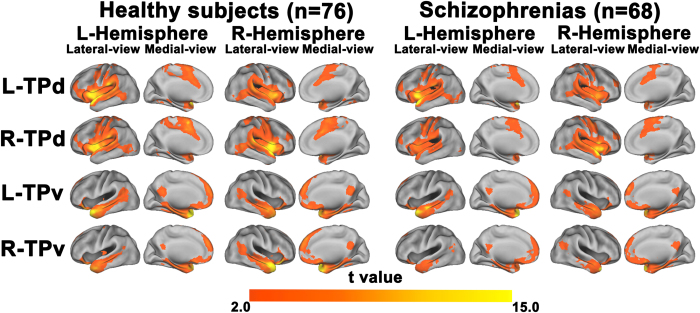
The resting-state functional connectivity (rsFC) map of each subregion of the temporal pole (TP) for each group. Only the positive rsFC map of each TP subregion is depicted for each group. All of the images were thresholded at two-tailed p < 0.05, with false discovery rate correction and cluster size > 30 voxels. L, left; R, right; TPd, dorsal subregion of the TP; TGv, ventral subregion of the TP.

The rsFC map of each TP subregion for each group is depicted in Fig. 2. In the healthy comparison subjects, each TP subregion had a specific rsFC pattern. The TPd was mainly connected to the language areas around the Sylvian fissure and the sensorimotor cortex, which is consistent with a previous study^25^. The TPv had connectivity to the middle temporal gyrus (MTG), medial prefrontal cortex (MPFC), and posterior cingulate cortex (PCC). The latter two regions are critical nodes in the default-mode network (DMN). The overlap maps between each TP subregion and “canonical” RSNs in the healthy comparison subjects are shown in Figure S7 in Supplement 1. We found that 63.1% of voxels in the TPd were in the auditory network, 23.5% of voxels in the TPd overlapped with the salience network, 3.1% of voxels in the TPv were in the auditory network, and 8.2% of voxels in the TPv overlapped with the DMN. Compared to the healthy comparison subjects, schizophrenia patients showed similar rsFC patterns, but with a smaller spatial extent.

### Between-group differences in rsFC in the TP subregions

**Figure 3:**
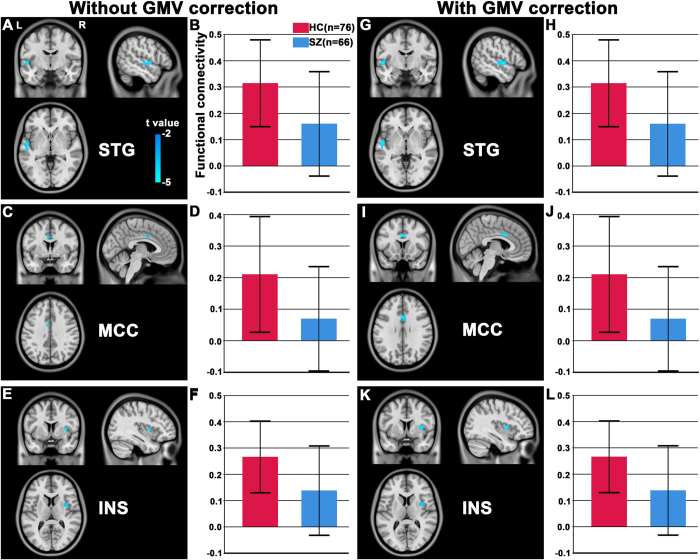
Altered resting-state functional connectivity (rsFC) in the dorsal subregion (TPd) of the right temporal pole in the schizophrenia patients. Compared to the healthy comparison subjects (HC), the schizophrenia patients (SZ) exhibited decreased rsFC between the right TPd and (**A,G**) the left superior temporal gyrus (STG), (**C,I**) left posterior mid-cingulate cortex (MCC) and (**E,K**) right insular cortex (INS). (**A,C,E**) shows the results without grey matter volume (GMV) correction and (**G,I,K**) depicts the results with GMV correction. The rsFC intensity of each cluster is shown in (**B,D,F**, without GMV correction) and (**H,J,L**, with GMV correction). Error bars represent the SD. L, left; R, right.

Among the 4 TP subregions, only the right TPd exhibited a significant difference in rsFC between the schizophrenia patients and healthy comparison subjects (Table 2, Fig. 3A–F). Compared to the healthy comparison subjects, schizophrenia patients had reduced rsFC between the right TPd and the left superior temporal gyrus (STG), left posterior mid-cingulate cortex (pMCC), and right insular cortex (p < 0.05, two-tailed, FDR-corrected, cluster size > 30 voxels).

### Volumetric reduction of the TP subregions in schizophrenia

Compared to the healthy comparison subjects, the schizophrenia patients had significantly reduced GMV (Bonferroni p < 0.05, two-tailed) in the left TPd (reduction rate: 7.1%, p < 0.001), right TPd (reduction rate: 8.3%, p < 0.001) and right TPv (reduction rate: 3.3%, p = 0.014) subregions (Table S5 in Supplement 1).

### rsFC differences in the TP subregions after correction for confounding factors

To investigate the volumetric reduction effect on rsFC differences between groups, the rsFC analysis was repeated after the mean normalized GMV of the right TPd of each subject was added as a no-interest covariate. All three clusters derived from the rsFC analysis without GMV correction remained significant after GMV correction (Fig. 3G–L), suggesting that rsFC alterations in the right TPd were relatively independent characteristics in schizophrenia and not a result of volumetric reduction. Because there was a significant intergroup difference in tSNR in the left TPv, we re-performed the intergroup comparison of the rsFC of the left TPv with individual rSNR as an additional no-interest covariate. In contrast to our initial findings, we did not find any significant group differences in the rsFC of the left TPv.

### Between-group differences in the degree of nodes in the TP subregions

Compared to the healthy comparison subjects, the schizophrenia patients displayed nodes with significantly smaller degrees only in the right TPd, using all of the tested connectivity thresholds (r = 0: t = 2.204, p = 0.029; r = 0.1: t = 2.446, p = 0.016; r = 0.2: t = 2.218; p = 0.028; r = 0.3: t = 2.098, p = 0.038; and r = 0.4: t = 2.006, p = 0.047) (Figure S8 in Supplement 1). However, we did not find any significant intergroup differences in the degree of nodes in any other TP subregion (p > 0.05, Bonferroni correction).

### Validation of group differences in rsFC

Because the TP subregions extracted from the maximal probability maps may result in overlap across subregions due to the normalization and smoothing, we also adopted another method (spheres with a radius of 6 mm centred at the centre of gravity of each TP subregion) to define the seeds of these TP subregions. We repeated our analyses and found that the right TPd was the only subregion that demonstrated significant rsFC differences between the schizophrenia patients and healthy comparison subjects (Figure S9 in Supplement 1). The locations of these clusters largely overlapped with those derived from the method using the maximal probability maps.

### Correlations between the rsFC of the TP subregions and clinical parameters

**Figure 4:**
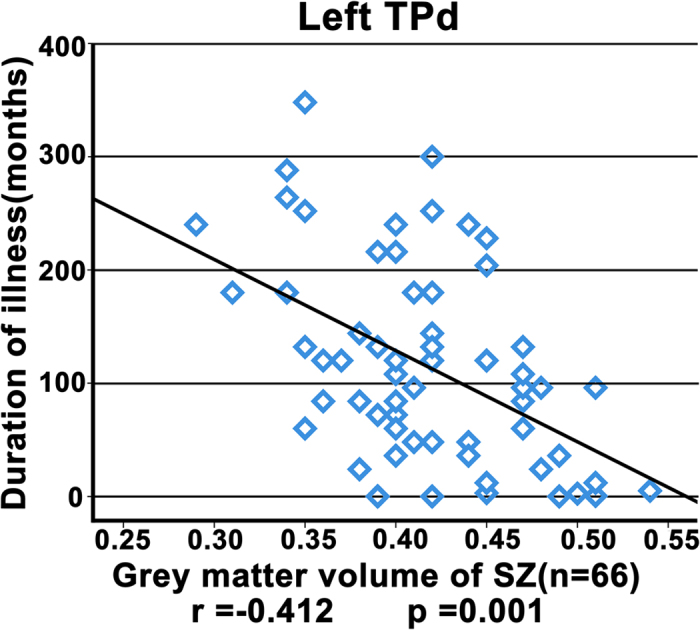
Correlation between grey matter volume (GMV) in the dorsal subregion (TPd) of the left temporal pole and the duration of illness in the schizophrenia patients. The p value remained significant after Bonferroni correction.

In patients with schizophrenia, we did not find any significant correlations between rsFC of the right TPd and any of the clinical parameters, including PANSS score, duration of illness, and current antipsychotic dosage in chlorpromazine equivalents. However, GMV in the left TPd was negatively correlated with the duration of illness (Spearman’s rho = −0.412, p = 0.001, two-tailed) (Fig. 4). No significant correlations were found between the GMV of any of the TP subregions and PANSS score or antipsychotic dosage.

## Discussion

In this study, we used a modified fMRI technique to investigate changes in the rsFC of TP subregions in schizophrenia. We found that the schizophrenia patients exhibited significant rsFC and node degree changes only in the right TPd. Among the TP subregions, the right TPd also had the most prominent GMV reduction in these patients. These findings suggest a selective impairment of TP subregions in schizophrenia. Specifically, the right TPd exhibited reduced rsFC with brain regions involved in language processing and multisensory integration, which may account for the relevant functional deficits of schizophrenia.

The majority of resting-state fMRI studies have used EPI sequences to acquire functional imaging data; however, the TP is one of the brain regions frequently subjected to susceptibility-induced signal loss and distortion during EPI scans. In this study, we adopted a SENSE-SPIRAL sequence to acquire resting-state fMRI data; this sequence has been confirmed to be superior to the EPI sequence in terms of imaging quality^27^. Spiral sampling of k-space has reduced sensitivity to motion, shortened readout time, improved signal intensity, and reduced geometric distortion^36^. SENSE is a parallel imaging method and has been used to shorten scan time and to reduce susceptibility-induced artefacts^37^. By combining the two techniques, the SENSE-SPIRAL sequence is especially suitable for imaging brain regions that are apt to have susceptibility-induced artefacts^27^. We found that the fMRI data acquired using the SENSE-SPIRAL sequence exhibited less signal loss in the medial TP and less distortion in the TP and orbitofrontal regions compared with the fMRI data acquired using the EPI sequence. Consequently, the fMRI data acquired using the SENSE-SPIRAL sequence were used for rsFC analyses in the present study.

Consistent with previous findings of GMV reduction in the TP^9^,^10^,^11^,^12^, we further revealed that volumetric reduction was more prominent in the dorsal subregion of the TP in schizophrenia. Moreover, the GMV of the left dorsal subregion was negatively correlated with the duration of illness, suggesting that neurodegenerative mechanisms may be at least partly related to the TP pathology in schizophrenia. Although the functional implications of the selective structural damage in the TP remain unclear, it may be related to clinical manifestations; this hypothesis has been at least partially supported by previous studies that revealed correlations between TP volume and the severity of formal thought disorder^14^ and abnormal theory of mind^15^ in schizophrenia.

The most important finding of our study is that only the right dorsal TP exhibited significant functional disconnection in schizophrenia. This selective functional disconnection remained significant even after GMV correction and was not correlated with the duration of illness or with antipsychotic dosage, suggesting that functional disconnection of the right dorsal TP is a characteristic of schizophrenia, which may result from the abnormal neurodevelopment in schizophrenia. Abnormal activation has frequently been reported in the dorsal TP in schizophrenia patients with auditory hallucination^38^,^39^, although this is not direct evidence of the selective functional disconnection of the TP. Consistent with previously reported rsFC patterns in TP subregions^25^,^26^, we found that the dorsal TP was mainly connected to auditory, somatosensory, motor, and language-related brain regions. This connectivity pattern supports the idea that the dorsal TP may play an important role in language, multisensory integration, and sensorimotor integration. Therefore, reduced rsFC of the right dorsal TP with the insular cortex, STG, and pMCC may underlie these functional deficits in schizophrenia.

Patients with schizophrenia often display language impairment, especially at the level of sentences and discourse^40^,^41^. The right dorsal TP participates in multiple language functions, including idiom comprehension, non-metric rhythm, phonological learning, and voice recognition^42^,^43^,^44^. Functional disconnection of the right dorsal TP from additional language-related regions (the insular and STG) may underlie the impairment of these higher-order language functions in schizophrenia patients. The middle longitudinal fascicle connects the dorsal TP and STG to the angular gyrus; its integrity may be related to language processing^45^. Patients with chronic schizophrenia exhibit significant damage in white matter integrity in this tract^46^; therefore, anatomical disconnection may underlie the functional disconnection of the dorsal TP in schizophrenia. The dorsal TP is involved in multisensory integration^4^,^5^, and the STG, pMCC, and insular cortex are also implicated in multisensory or sensorimotor integration^47^,^48^,^49^. Therefore, the functional disconnection of the right dorsal TP from the STG, pMCC, and insular cortex may reflect multisensory or sensorimotor integration deficits in schizophrenia patients^50^,^51^.

There are several limitations of our study. First, no correlations were found between the imaging data and the clinical data, which makes our interpretation less reasonable. However, PANSS score is a type of state index; the reduced GMV and decreased rsFC observed in our study may be a trait of schizophrenia. Second, although we did not find any associations between drug dosage and altered functional connectivity in the schizophrenia patients, the majority of these patients have received mediation for a long time, and the effect of medication was probably not dose-independent. Thus, first-episode antipsychotic-naïve schizophrenia patients with more homogeneous clinical features should be included in future studies. Finally, we defined the TP subregions according to previous parcellations of the TP based on anatomical connection patterns^25^. It seems that defining the TP subregions based on rsFC patterns may be more reasonable for the rsFC comparisons. However, the only parcellation study of the TP based on rsFC patterns was restricted to the left TP^26^, from which we cannot extract the right TP subregions. Although our definition of the TP subregions may not be the best one, the dorsal and ventral subdivisions of the TP are consistent with both of the TP parcellation studies^25^,^26^.

In conclusion, we used an improved fMRI technique to detect resting-state functional connectivity alterations in TP subregions in schizophrenia. The alterations were characterized by selective functional disconnection of the dorsal TP and brain regions involved in multisensory integration and higher-level language processing. Future studies that combine functional connectivity with the assessment of phenotypes more specific to these two functions may help elucidate the functional implications of functional disconnection of the dorsal TP in schizophrenia.

## Additional Information

**How to cite this article**: Xu, L. *et al*. Selective Functional Disconnection of the Dorsal Subregion of the Temporal Pole in Schizophrenia. *Sci. Rep*. **5**, 11258; doi: 10.1038/srep11258 (2015).

## Supplementary Material

Supplementary Information

## Figures and Tables

**Table 1:** Demographic and clinical characteristics of schizophrenia patients and healthy comparison subjects.

**Characteristics**	**Healthy Comparison Subjects (n = 76)**		**Schizophrenia Patients (n = 66)**		**Analysis**	
	**Mean**	**SD**	**Mean**	**SD**	**t/χ**^**2**^	**p**
Age (years)	33.0	10.4	33.0	7.6	t = −0.026	0.980
Sex	M38	F38	M38	F28	χ^2^ = 0.815	0.367
Duration of illness (months)	—	—	114.0	87.7	—	—
Positive and Negative Syndrome Scale score						
Positive subscore	—	—	17.0	8.1	—	—
Negative subscore	—	—	21.1	8.7	—	—
General subscore	—	—	72.6	23.3	—	—
Current antipsychotic dosage (chlorpromazine equivalents) (mg/d)	—	—	437.4	336.2	—	—

**Table 2:** Functional connectivity changes in the right dorsal subregion of the temporal pole in schizophrenia patients.

Brain Regions^a^	**Montreal Neurological Coordinates (x, y, z)**	**Peak t Value**	**Cluster Size (Voxels)**^b^
Left superior temporal gyrus	−51,6,0	−4.66	53
Left posterior mid-cingulate cortex	−6,0,36	−4.54	43
Right insular cortex	33,3,9	−4.30	33

^a^Significant changes were only observed in schizophrenia patients < healthy comparison subjects, with a false discovery rate-corrected, two-tailed p < 0.05 and cluster size > 30 voxels.

^b^Voxel size was 3 × 3 × 3 mm^3^.
