# Supplementary material for: Selective Functional Disconnection of the Dorsal Subregion of the Temporal Pole in Schizophrenia
Source: Sci Rep. 2015 Jun 9;5:11258. doi: 10.1038/srep11258 (PMC4460906; doi:10.1038/srep11258)
Supplement: Supplementary Information [file srep11258-s1.pdf]

Full Title:

Selective Functional Disconnection of the Dorsal Subregion of the Temporal Pole in Schizophrenia

Lixue Xu [1, 4], Wen Qin [1, 4], Chuanjun Zhuo [1, 2, 3, 4], Jiajia Zhu [1], Huaigui Liu [1], Xingyun Liu [1], Yongjie Xu [1], and Chunshui Yu*, [1]

Running Title:

Selective Functional Disconnection of the TP Subregions in Schizophrenia

---

[1] Department of Radiology and Tianjin Key Laboratory of Functional Imaging, Tianjin Medical University General Hospital, Tianjin 300052, China

[2] Tianjin Anding Hospital (Tianjin Mental Health Center), Tianjin City 300222, China

[3] Tianjin Anning Hospital, Tianjin City 300300, China

[4] These authors contributed equally to the article.

***Address correspondence to:** Dr. Chunshui Yu.

Department of Radiology, Tianjin Medical University General Hospital, No. 154, Anshan Road, Heping District, Tianjin 300052, China.

E-mail: chunshuiyu@vip.163.com

Supplementary Figures and Tables

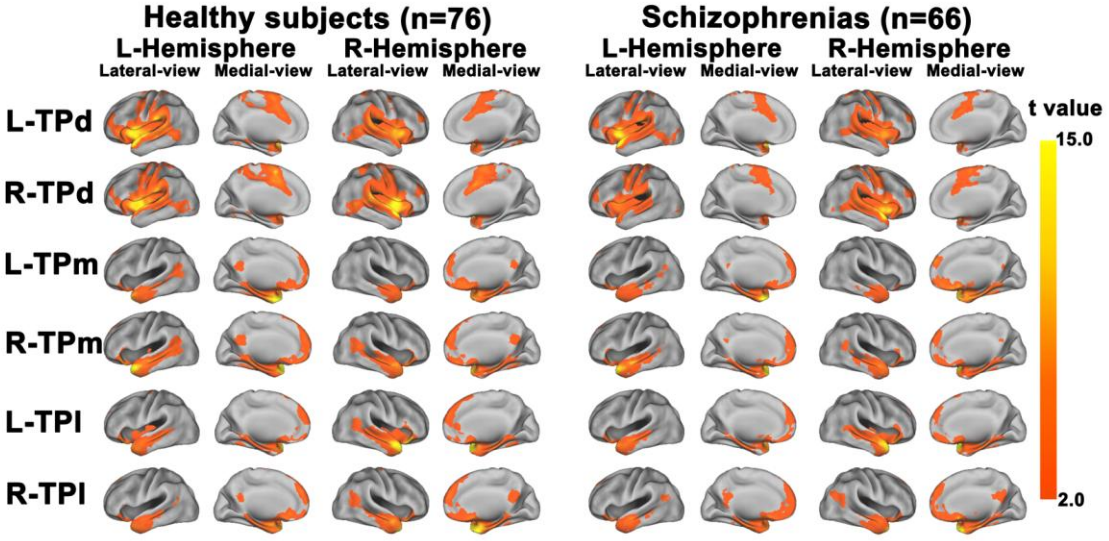

Figure S1: The resting-state functional connectivity map of each subregion (3 subregions each side) in the temporal pole of each group. Only the positive resting-state functional connectivity map of each subregion in the temporal pole of each group is depicted. All the images were thresholded at a two-tailed p < 0.05, false discovery rate correction, cluster size > 30 voxels. The lateral subregions shared the similar rsFC pattern with medial subregions. L, left; R, right; TPd, dorsal subregion of the temporal pole; TPl, lateral subreigon of the temporal pole; TPm, medial subregion of the temporal pole.

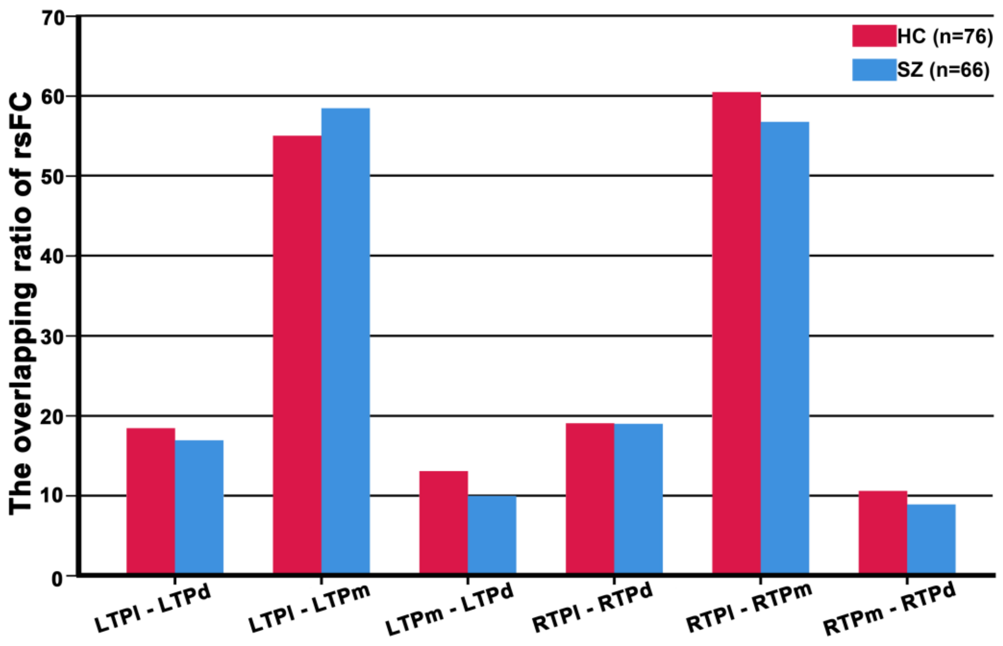

Figure S2: The overlapping ratios of resting-state functional connectivity (rsFC) between subregions of the temporal pole. The overlapping ratios between bilateral TPd and TPm were much higher. L, left; R, right; TPd, dorsal subregion of the temporal pole; TPl, lateral subreigon of the temporal pole; TPm, medial subregion of the temporal pole.

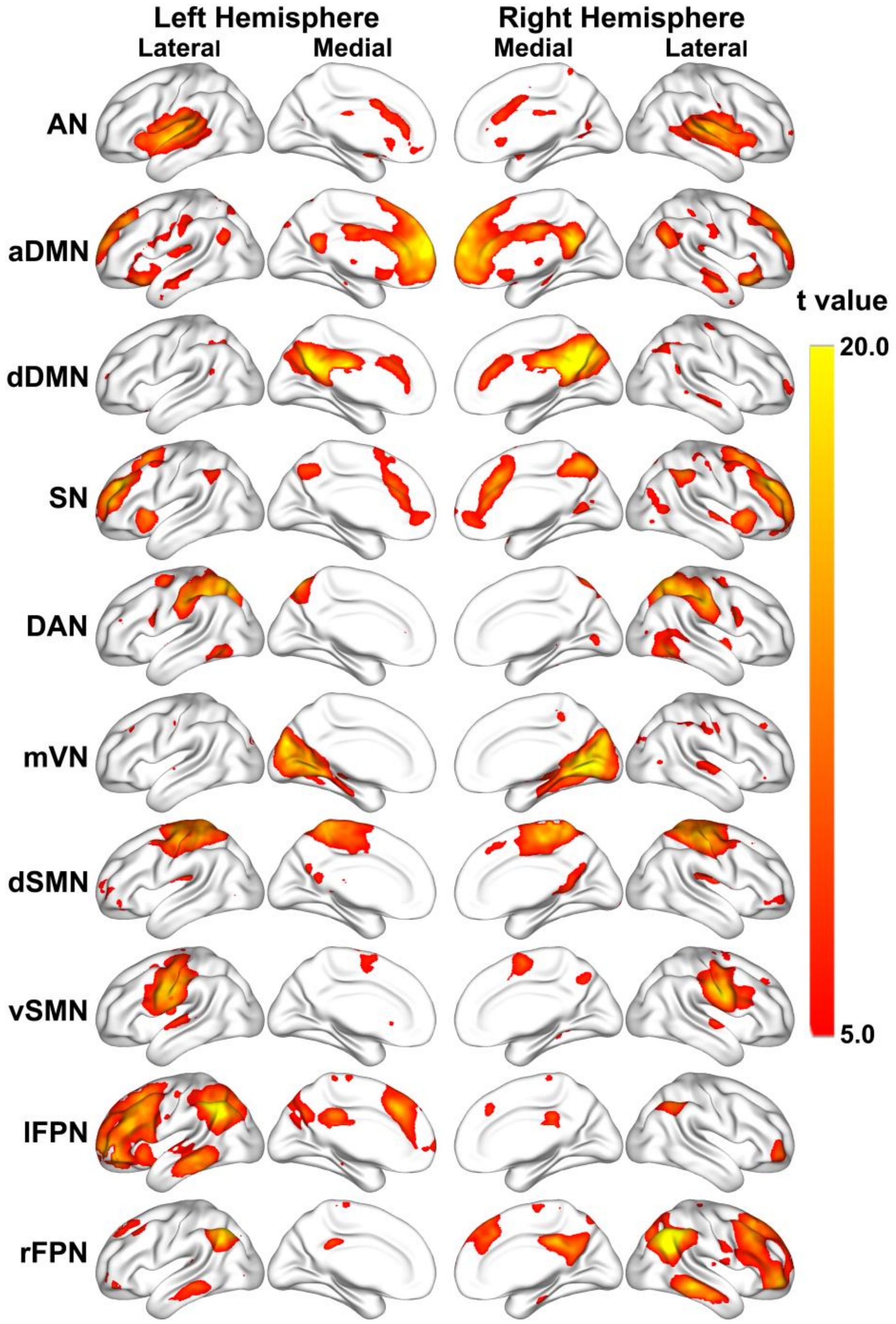

Figure S3: Spatial maps of the resting state networks (RSNs) identified by independent component analysis. AN, auditory network; aDMN, anterior default mode network; pDMN, posterior default mode network; SN, salience network; DAN, dorsal attention network; mVN, medial visual network; dSMN, dorsal sensorimotor network; vSMN, ventral sensorimotor network; lFPN, left frontoparietal network; rFPN, right frontoparietal network.

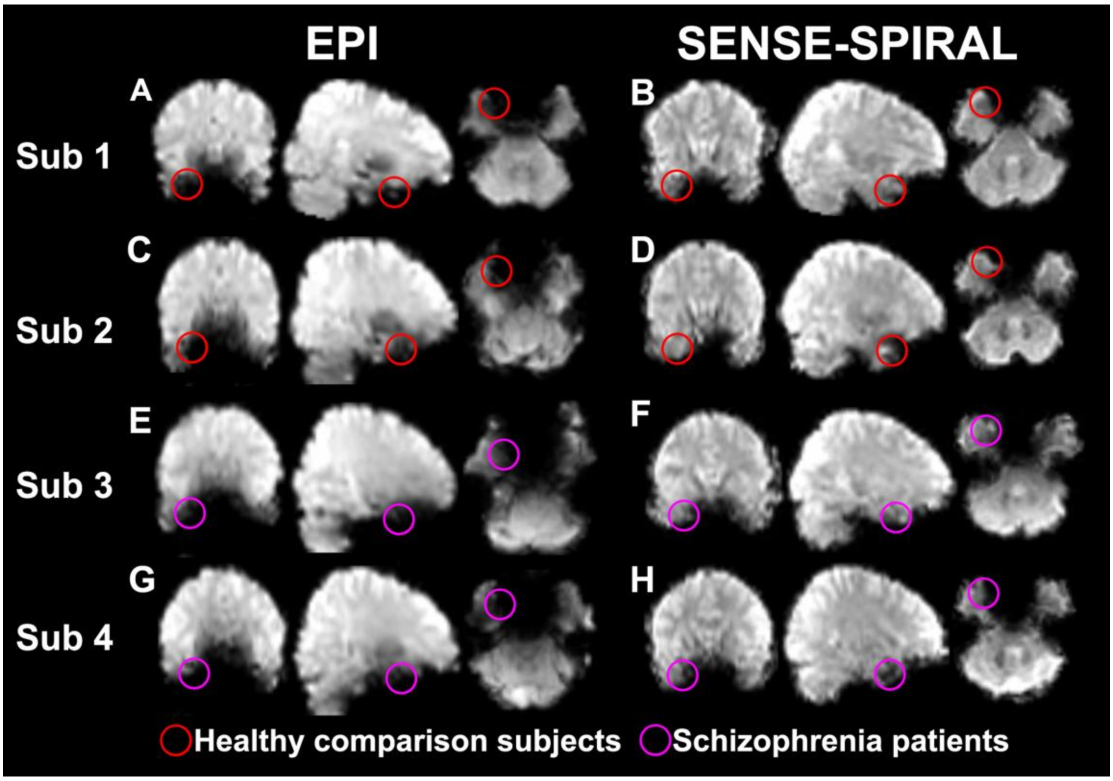

Figure S4: Signal intensity in the TP regions. Compared to the EPI sequence (A, C, E, G), the SENSE-SPIRAL sequence (B, D, F, H) improves signal intensity in the TP regions. The TP region is marked using a circle. Images of each row are derived from a single subject. Subject 1 and 2 are healthy comparison subjects, whereas subject 3 and 4 are schizophrenia patients.

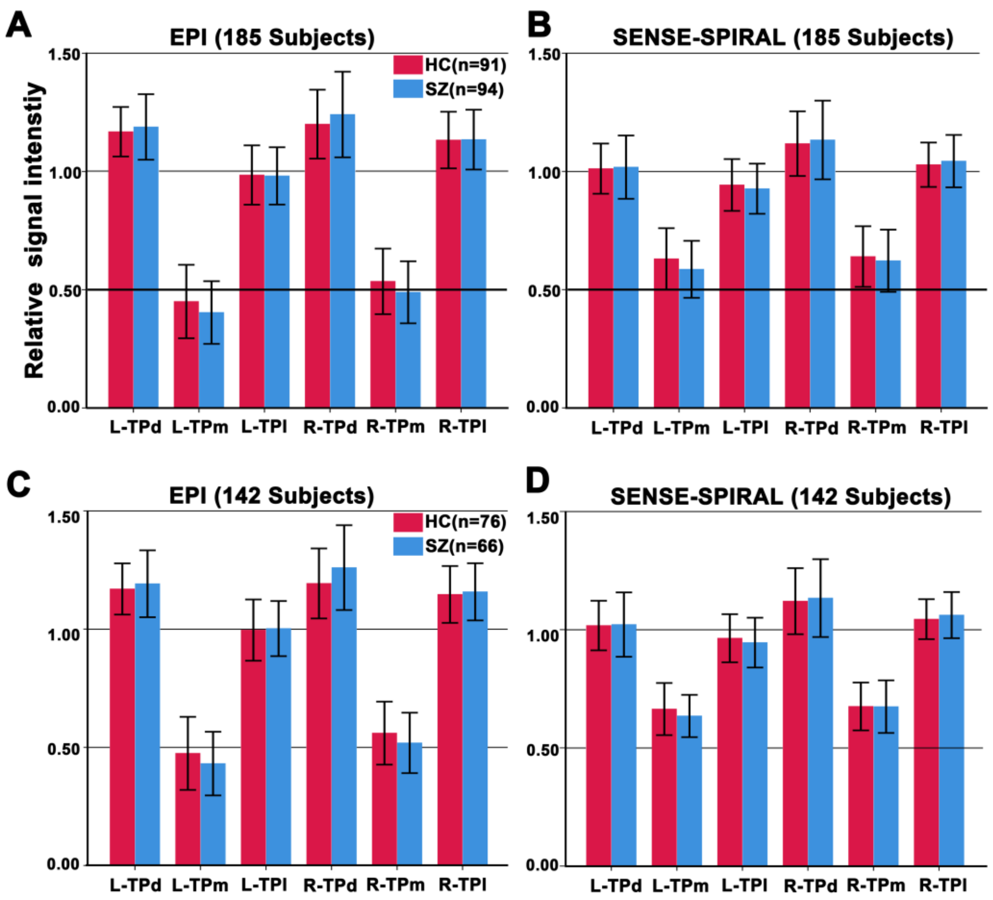

Figure S5: The mean relative signal intensity (rSI) of each subregion in the temporal pole. The mean rSI of each subregion of all the 185 subjects derived from the single-shot echoplanar imaging (EPI) sequence is shown in (A). (B) shows the rSIs derived from the sensitivity-encoded spiral-in imaging (SENSE-SPIRAL) sequence. The horizontal line indicates that the rSI is 0.5. The mean rSIs of all the subregions derived from the SENSE-SPIRAL sequence were higher than 0.5, whereas the mean rSI in the left medial part of the temporal pole acquired by the EPI sequence was lower than 0.5. After excluding subjects whose rSI in any region was lower than 0.5, 142 subjects remained. (C) reflects the mean rSI in each subregion of 142 subjects which derived from the EPI sequence, and (D) shows those from the SENSE-SPIRAL sequence. Error bars represent the SD. TPd, dorsal subregion of the temporal pole; TPl, lateral subreigon of the temporal pole; TPm, medial subregion of the temporal

pole.

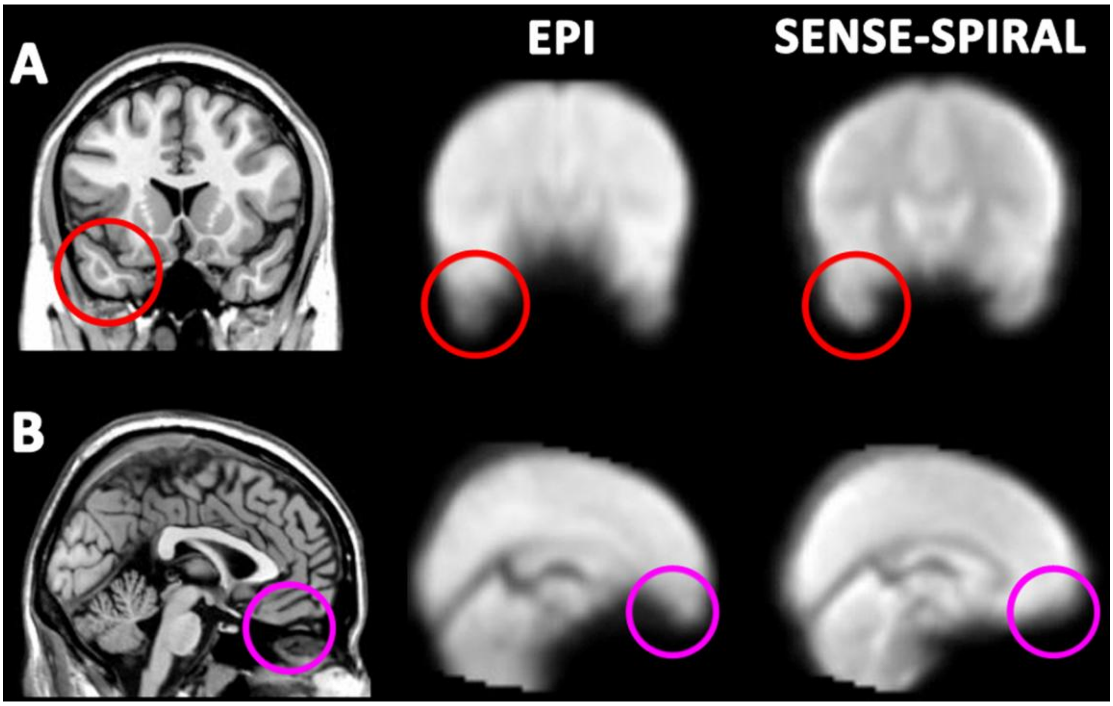

Figure S6: Distortion of functional images derived from various acquisition sequences. Coronal and sagittal functional images show that (A) the temporal pole and (B) orbitofrontal cortex exhibit less distortion in fMRI data acquired by the sensitivity-encoded spiral-in imaging (SENSE-SPIRAL) sequence than in those acquired by the single-shot echo planar imaging (EPI) sequence.

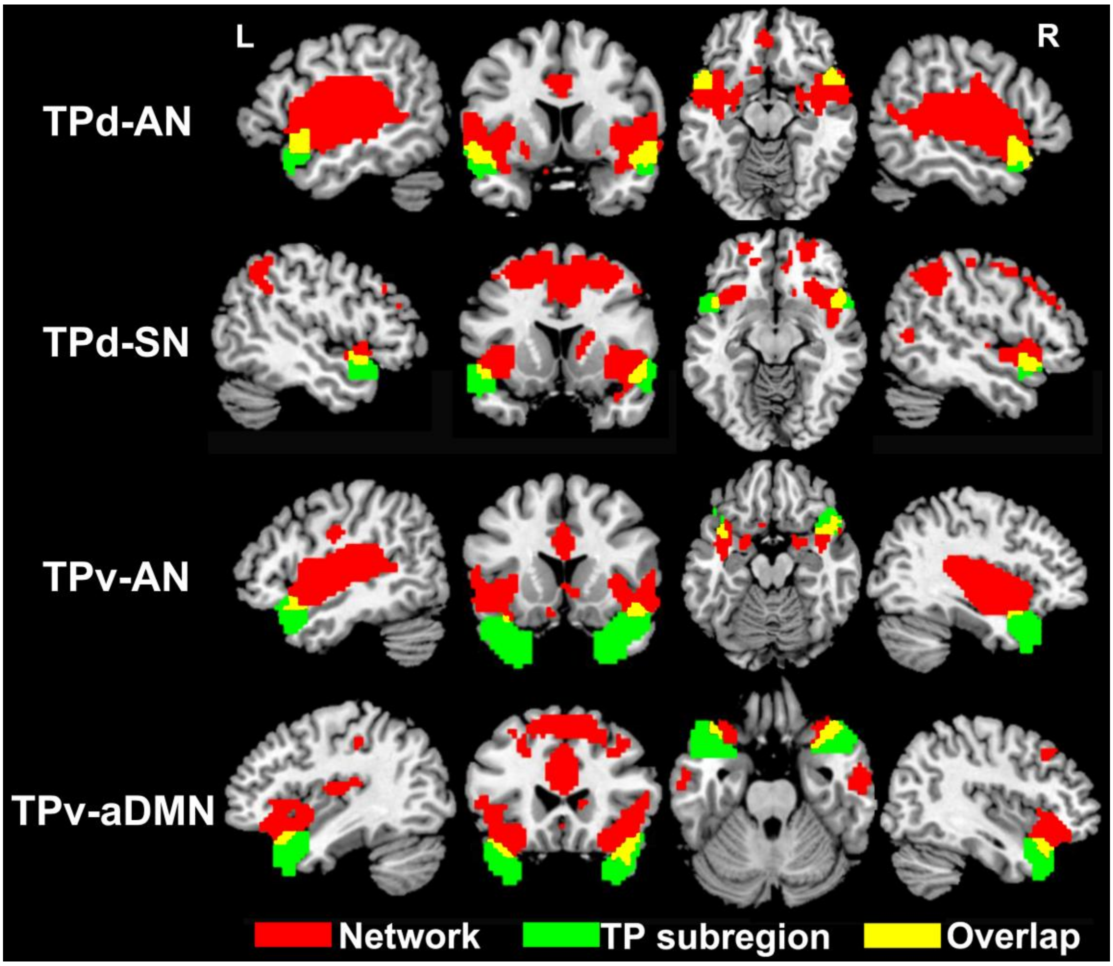

Figure S7: The overlap between the resting-state networks (RSNs) and TP subregions. Red color represents each RSN; green color denotes each TP subregion; and yellow color indicates the overlapping area. L, left; R, right.

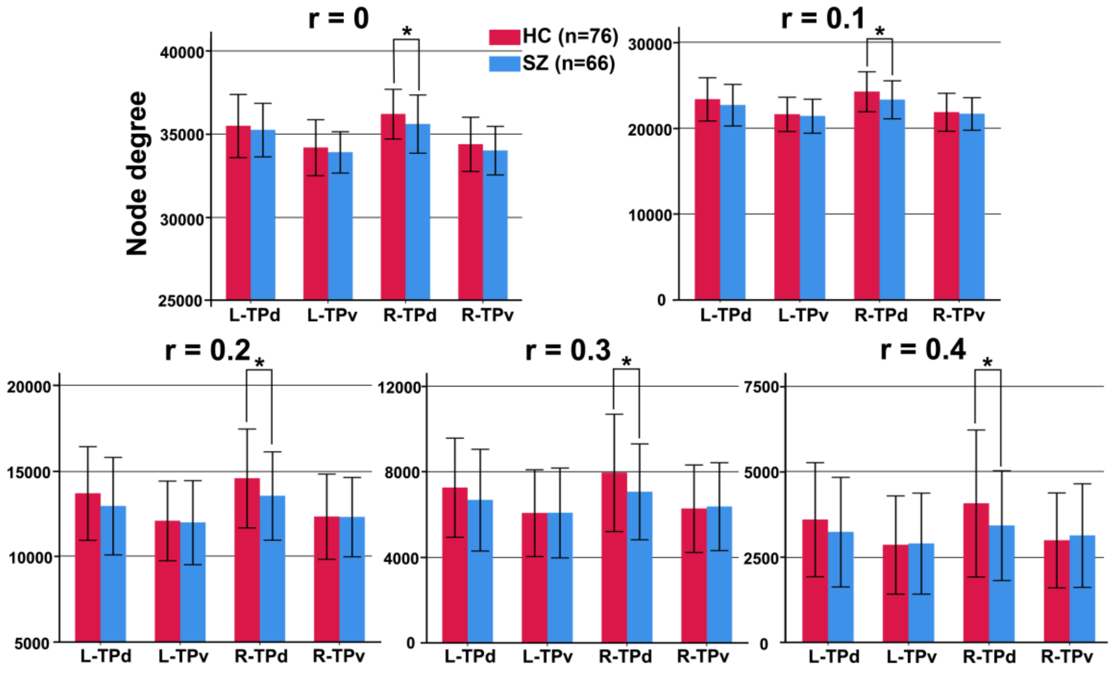

Figure S8: Group differences in node degree in each TP subregion under different connectivity thresholds (r = 0, 0.1, 0.2, 0.3 and 0.4). Only the right TPd displays significantly decreased node degree in schizophrenia patients than in healthy comparison subjects ($p < 0.05$, Bonferroni correction). Error bars represent the SD. TPd, dorsal subregion of the temporal pole; TPv, ventral subregion of the temporal pole.

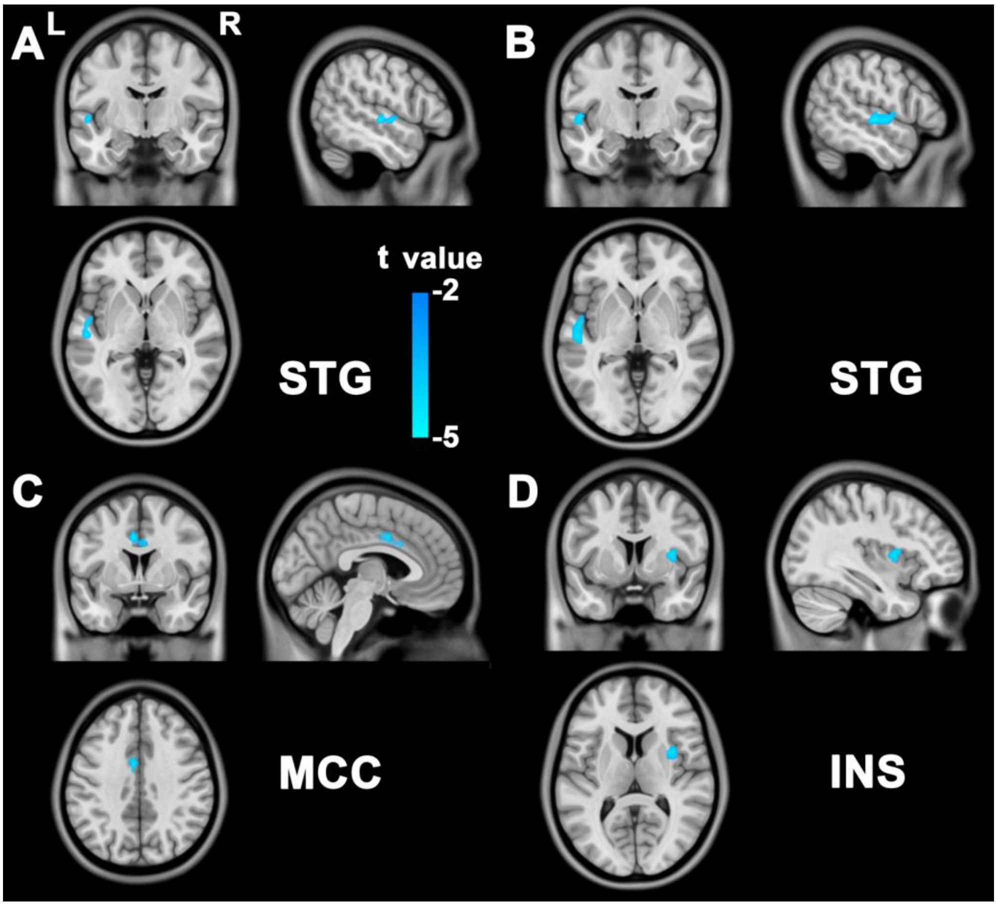

Figure S9: Altered resting-state functional connectivity in the right dorsal subregion (TPd) of the temporal pole in schizophrenia patients (n = 66) using spheric regions of interest. Using spheric regions of interest as seed regions, schizophrenia patients exhibited decreased functional connectivity in the right TPd with the (A) left superior temporal gyrus (STG). To observe the group differences at the same voxel level as the original results which regarded maximal probability templates as seed regions, images were thresholded at p value of 0.0006 (the same p value of the original results by false discovery rate correction, $p < 0.05$, two-tailed). Weakened rsFC in the right TPd were observed with the (B) left STG, (C) left mid-cingulate cortex (MCC), and (D) right insula (INS) when setting the threshold at two-tailed uncorrected $p < 0.005$, cluster size>30 voxels.

Table S1: The mean relative signal intensity (rSI) of each TP ROI of all subjects (n = 185).

Regions	Mean rSI (n = 185)						Subject counts for rSI < 0.5[c]	
	EPI		SENSE-SPIRAL		Analysis[a]		EPI	SENSE-SPIRAL
	Mean	SD	Mean	SD	t values	p values[b]		
Left TPd	1.178	0.123	1.015	0.121	**16.744**	< **0.001**	1/185	0/185
Left TPm	0.426	0.146	0.608	0.127	**-17.585**	< **0.001**	136/185	36/185
Left TPl	0.983	0.123	0.935	0.108	**5.030**	< **0.001**	0/185	0/185
Right TPd	1.221	0.165	1.125	0.152	**7.772**	< **0.001**	0/185	0/185
Right TPm	0.512	0.137	0.631	0.130	**-11.351**	< **0.001**	87/185	25/185
Right TPl	1.134	0.123	1.036	0.103	**11.135**	< **0.001**	0/185	0/185

[a] To compare the differences between relative signal intensity acquired by EPI and SENSE-SPIRAL sequence, we performed a paired *t*-test.

[b] These p values (two-tailed) remained significant after Bonferroni correction.

[c] The counts for subjects whose relative signal intensity was less than 0.5 are presented by dividing the total subjects number (n = 185).

TPd, dorsal subregion of the temporal pole; TPm, medial subregion of the temporal pole; TPl, lateral subregion of the temporal pole.

Table S2: The mean relative signal intensity (rSI) of each TP ROI of the selected subjects (n = 142).

Regions	Mean rSI (n = 142) from the SENSE-SPIRAL sequence		
	Mean	SD	Range
Left TPd	1.019	0.120	0.710-1.385
Left TPm	0.651	0.102	0.500-1.104
Left TPl	0.955	0.103	0.738-1.291
Right TPd	1.127	0.151	0.682-1.463
Right TPm	0.675	0.106	0.500-0.987
Right TPl	1.052	0.091	0.801-1.326

TPd, dorsal subregion of the temporal pole; TPm, medial subregion of the temporal pole; TPl, lateral subregion of the temporal pole.

Table S3: Group differences in the relative signal intensity (rSI) of the sensitivity-encoded spiral imaging (SENSE-SPIRAL) data.

Regions	HC (n = 76)		SZ (n = 66)		Analysis[a]	
	Mean	SD	Mean	SD	t value	p value
Left TPd	1.017	0.105	1.022	0.136	-0.205	0.838
Left TPv	0.770	0.087	0.755	0.087	1.108	0.270
Right TPd	1.120	0.140	1.133	0.165	-0.512	0.610
Right TPv	0.844	0.077	0.856	0.086	-0.858	0.392

[a] To compare the differences in the rSI acquired by SENSE-SPIRAL between groups, we performed a two-sample *t*-test.

HC, healthy comparison subjects; SZ, schizophrenia patients; TPd, dorsal subregion of the temporal pole; TPm, medial subregion of the temporal pole; TPl, lateral subregion of the temporal pole.

Table S4: Group differences of the temporal spatial-noise ratio (tSNR) in each TP subregion.

Regions	HC		SZ		Analysis [a]	
	Mean	SD	Mean	SD	t value	p value
Left TPd	35.983	8.815	33.485	6.578	1.889	0.061
Left TPv	26.106	5.577	24.139	3.950	**2.390**	**0.018** [b]
Right TPd	41.149	10.766	38.111	8.705	1.831	0.069
Right TPv	29.268	6.456	28.138	5.277	1.130	0.260

[a] To compare the differences in the tSNR between healthy comparison subjects and schizophrenia patients, we performed a two-sample *t*-test.

[b] The p value (two-tailed) remained significant after Bonferroni correction.

HC, healthy comparison subjects; SZ, schizophrenia patients; TPd, dorsal subregion of the temporal pole; TPm, medial subregion of the temporal pole; TPl, lateral subregion of the temporal pole.

Table S5: Relative grey matter volume (GMV) of each subregion in the temporal pole.

	Left TPd	Left TPv	Right TPd	Right TPv
Relative GMV of HC (n=76)	0.531	0.505	0.540	0.553
Relative GMV of SZ (n=66)	0.493	0.489	0.495	0.529
Chang of GMV (%)	-7.126	-3.337	-8.338	-6.575
F values	**13.676**	3.665	**15.078**	**6.146**
p values	**< 0.001**[a]	0.058	**< 0.001**[a]	**0.014**[a]

[a] Group differences in GMV were calculated by a general linear model, with sex and age being covariates of no interest. These p values remained significant after Bonferroni correction.

HC, healthy comparison subjects; SZ, schizophrenia patients; TPd, dorsal subregion of the temporal pole; TPm, medial subregion of the temporal pole; TPl, lateral subregion of the temporal pole.
